# Crystal structure reveals conservation of amyloid-β conformation recognized by 3D6 following humanization to bapineuzumab

**DOI:** 10.1186/alzrt261

**Published:** 2014-06-02

**Authors:** Hadar Feinberg, José W Saldanha, Linnea Diep, Amita Goel, Angela Widom, Geertruida M Veldman, William I Weis, Dale Schenk, Guriqbal S Basi

**Affiliations:** 1 Elan Pharmaceuticals, Inc. 300 Technology Sq., Cambridge, MA 02139, USA; 2 Departments of Structural Biology and of Molecular & Cellular Physiology, 299 Campus Drive, Stanford University School of Medicine, Stanford, CA 94305, USA; 3 Pfizer, Cambridge, MA, USA; 4 Prothena Biosciences, Inc., 650 Gateway Blvd., San Francisco, CA 94080, USA; 5 National Institute for Medical Research, The Ridgeway, Mill Hill, London NW7 1AA, UK; 6 Abbvie Bioresearch Center, Worcester, MA, USA

## Abstract

**Introduction:**

Immunotherapy targeting amyloid-β peptide is under active clinical investigation for treatment of Alzheimer’s disease (AD). Among the hypotheses being investigated for impact on clinical outcome are the preferred epitope or conformation of amyloid-β to target for treatment, and the mechanism of action underlying immunotherapy. Bapineuzumab (humanized 3D6), a neo-epitope specific antibody recognizing amyloid-β1-5 with strong preference for an exposed Asp residue at the N-terminus of the peptide, has undergone advanced clinical testing for treatment of AD.

**Methods:**

To gain further insight into the epitope conformation, we interrogated structural details of amino-terminal epitopes in amyloid-β using x-ray crystallography of 3D6Fab:amyloid-β complexes. Humanization of 3D6 was carried out using standard procedures integrating recombinant methods, sequence informatics, and homology modeling predictions to identify important mouse framework residues for retention in the finished humanized product.

**Results:**

Here we report the crystal structure of a recombinant Fab fragment of 3D6 in complex with amyloid-β1-7 solved at 2.0 Å resolution. The N-terminus of amyloid-β is bound to 3D6 as a 3_10_ helix. The amino-terminal Asp residue is buried deepest in the antibody binding pocket, with the Cβ atom of residue 6 visible at the entrance to the binding pocket near the surface of the antibody. We further evaluate homology model based predictions used to guide humanization of 3D6 to bapineuzumab, with actual structure of the Fab. The structure of the Fab:amyloid-β complex validates design of the humanized antibody, and confirms the amyloid-β epitope recognized by 3D6 as previously mapped by ELISA.

**Conclusions:**

The conformation of amyloid-β antigen recognized by 3D6 is novel and distinct from other antibodies recognizing N-terminal epitopes. Our result provides the first report demonstrating structural conservation of antigen contact residues, and conformation of antigen recognized, between the parent murine antibody and its humanized version.

## Introduction

Immunotherapy targeting amyloid beta (Aβ) peptide has been demonstrated to prevent or reverse a range of Alzheimer’s disease (AD) related pathologies, in both transgenic mouse models and AD patients [1-5]. Efficacy against Aβ related behavioral deficits has also been reported in transgenic mouse models of AD [6-9]. Despite the failure of initial efforts with immunotherapy to meet primary endpoints in pivotal clinical trials [10,11], the preponderance of successful pre-clinical studies targeting Aβ provide support for ongoing clinical trials with Aβ immunotherapy for treatment of AD in humans, evidenced by the multiplicity of approaches continuing clinical testing [12-15] (ClinicalTrials.gov Identifier: NCT01760005).

A number of important questions regarding efficacy following Aβ immunotherapy remain under investigation. These include: 1) the mechanisms of action; 2) preferred Aβ epitope to target; and 3) the specific form of Aβ recognized by a given antibody. In our hands, antibody isotypes with maximal effector function targeting amino-terminal epitopes provided the greatest efficacy across a number of end-points in preclinical studies [5,16,17]. Fine mapping the epitope specificity among antibody responders from an active vaccination phase 2 trial in AD patients immunized with AN1792 (Aβ1-42 peptide) revealed exquisite specificity for the amino-terminus of Aβ peptide [18], providing some clinical support for targeting the amino-terminus of Aβ for AD immunotherapy.

Among the efficacious *in vivo* amino-terminal epitope targeting antibodies we characterized and humanized for clinical development, the antibody 3D6 presented an attractive candidate due to its neo-epitope specificity for the amino-terminus of Aβ, namely a strong preference for a free Asp residue at position 1 of Aβ. This unique specificity of 3D6 precludes recognition of unprocessed amyloid precursor protein (APP) (hypothesized to be a desirable attribute in a clinical candidate), and is preserved in bapineuzumab (humanized 3D6, version 2), as reported below. Furthermore, the 3D6 epitope is detectable in all forms of Aβ tested [5], from compacted β-amyloid plaques in AD and platelet-derived growth factor (PDGF) promoter driven APP transgenic mouse model of AD (PDAPP) brain, to soluble oligomeric species. The latter are thought to be a primary mediator of neurotoxicity, and have also been postulated to underlie behavioral impairments in AD Tg mice [19]. In summary, the properties of 3D6 most closely reflected the antibody response mapped in AN1792 treated AD patients [18], supporting advancement of bapineuzumab (humanized 3D6v2) for clinical development [3,20-22].

To gain further insight into this specific Aβ epitope for immunotherapy of AD, we investigated antigen conformation recognized by different antibodies targeting amino-terminal epitopes of Aβ using X-ray crystallography of antibody:Aβ co-complexes solved to very high resolution (1.5 to 3 Å for all antibodies) [23]. Previously, we reported that three independently derived antibodies targeting Aβ residues 3–7 recognize antigen in an extended conformation along the surface of the antibody binding site [23]. Here we report that, in contrast, antibody 3D6, targeting Aβ residues 1–5, binds antigen in a 3_10_ helix. The antigen is bound by antibody such that the amino-terminus of the peptide is buried in a cleft in the antibody binding site, and the carboxy-terminus winds out to the surface of the antibody. Taken together, our findings reveal distinct conformations adopted by the amino-terminal epitope of Aβ, consistent with independent reports from other groups [24-26], and offer a testable hypothesis for the combinatorial manipulation of antibody activities targeting Aβ, for example, as bi-specific antibodies (wherein each arm of the Fab recognizes a different epitope/conformation of Aβ), incorporating the appropriate Fc isotype (for desired effector functions).

The high resolution crystal of 3D6 Fab + Aβ reported here, coupled with the structure of 4HIX (an early version of humanized 3D6 containing mouse framework residues deemed dispensable in bapineuzumab, see Results) in complex with Aβ [24] afforded the opportunity for retrospectively evaluating the humanization design of 3D6 leading to bapineuzumab. Comparison of our structure with 4HIX illustrates conservation of antigen conformation, and all features of antigen recognition by the antibody. This comparative analysis validates the design of bapineuzumab and attests to the robustness of antibody humanization technology as a platform for obtaining clinical development candidates from preclinical leads for disease therapy in humans.

## Methods

### Crystallization

Recombinant 3D6 Fab was expressed and purified from mammalian cell culture supernatant as previously described [23]. Crystallization conditions are summarized in Additional file 1: Table S1. Crystals of 3D6 Fab with either Aβ1-7 peptide or Aβ1-40 peptide were grown at 22 °C and frozen in liquid nitrogen for data collection.

### Data collection

Diffraction data for 3D6Aβ1-7 and 3D6Aβ1-40 were measured at 100 K at beamline 12–2 of the Stanford Synchrotron Radiation Lightsource. Data were processed with MOSFLM and SCALA [27]. Data statistics are summarized in Table 1.

**Table 1:** Crystallographic data statistics

	**3D6Aβ1-7**	**3D6Aβ1-40**
Space group	C2	P222_1_
Unit cell lengths (Å)	a = 126.8	a = 40.0
angles (°)	b = 69.4	b = 84.9
	c = 61.7	c = 175.9
	β = 115.4	
Resolution Å(last shell)	2.0 (2.05)	2.2 (2.26)
R_sym_(last shell)^a^	6.5 (23.0)	5.9 (18.9)
Mean((I)/σ(I))	15.0 (5.0)	19.9 (7.9)
% completeness (last shell)	100 (100)	100 (100)
Average multiplicity	3.8 (3.7)	5.8 (5.9)
Residues in final model (total)	1-218 (219)	1-204,
Light chain	1–132,	207–216 (219)
Heavy chain	140–219 (222)	1–100, 103–132,
		140–219 (222)
	1-6	
Aβ		1-5
R_free_ ^a^	20.4	22.7
R^a^	15.8	17.7
Average B (Å^2^)	28.5	29.4
Bond length RMSD (Å)	0.007	0.008
Angle RMSD (°)	1.1	1.2
Ramachandran plot: (% in preferred/ allowed/ outliers regions)^b^	97.2/2.8/0.0	95.8/3.9/0.3

^a^Rsym = ∑ _h_ ∑ _I_(| I_i_(h) - < I(h) > |) /∑h ∑ _i_I_i_(h) where I_i_(h) = observed intensity, and < I(h) > = mean intensity obtained from multiple measurements. R and R_free_ = ∑ h|F_o_(h) - F_c_(h)| /∑_h_F_o_(h), where F_o_(h) = observed structure factor amplitude and F_c_(h) = calculated structure factor amplitude for the working and test sets, respectively. ^b^As defined in Coot. RMSD, root mean square deviation of atomic position.

### Structure determination

Molecular replacement calculations were performed using the program COMO [28]. Molecular replacement was done in two stages, first finding the rotation and translation solution of the constant domain and later, while fixing the constant domain, finding the solution for the variable domain. Model building was done using the program Coot [29]. The structures were initially refined using Refmac [27] and at later stages using Phenix [30] with individual positional and B-factor refinement and five Translation Libration Screw (TLS) groups (the heavy and light chains were split into two groups each, at the hinge between the constant and the variable domains, and the peptide as a separate group).

In the final model of 3D6 + Aβ1-7 the side chain of residue 6 was not visible past Cβ. Only Aβ residues 1–5 are visible in the 3D6 + Aβ1-40 crystal structure. These crystals were grown in the 200 mM Zn^2+^, and several strong difference electron density peaks were modeled as Zn^2+^, based on the geometry of the surrounding ligands and the appearance of strong peaks at these positions in an anomalous difference map. One of these Zn^2+^ ions occupies the same spatial position as Aβ1-40 and hence cannot be present at this site at the same time as the peptide. The peptide and this Zn^2+^ ion were treated as alternative conformations each with 50% occupancy. The x-ray coordinates of the structures reported here have been deposited in the protein data base (pdb) and are accessible under the pdb identifiers 4ONF (Aβ1-7 complex) and 4ONG (Aβ1-40 complex).

### Affinity determination by Biacore

Interactions between anti-Aβ antibodies and biotinylated DAE10 peptide (Aβ1-10 conjugated to biotin at the carboxy-terminus and immobilized to streptavidin coated chips) were quantitatively analyzed using BIAcore plasmon resonance technology, and apparent kinetic rate constants were derived. Bio DAE-10 peptide was diluted in water at 1.0 mg/ml and kept at -80°C (stock). Following immobilization of the biotinylated DAE10 peptide, varying concentrations of each antibody were applied in triplicate and the binding was measured as a function of time. Affinity measurements chips were performed on chips coated at lower densities, typically 4 to 7 response units (RU) of the bio-peptide ligand. Antibodies were injected in triplicate at 100 nM at a flow rate of 30 μl/minute, injection time two minutes, post-injection time ten minutes, followed by regeneration of chips with 5 μl 0.1% tri-fluro acetic acid (TFA). All the antibodies were run in at least three different chips over three different density surfaces. From these measurements the apparent dissociation (kd) and association (ka) rate constants were derived and used to calculate a K_D_ value for the interaction, corrected for active concentration of antibody. Active concentration of antibody measurements was carried out using chips coated with bio-DAE peptide at high density. All the affinity data presented in this report were analyzed using the bivalent model.

### Humanization of 3D6

As the humanization of 3D6 preceded the elucidation of its crystal structure, the variable light and heavy regions of 3D6 were modeled using the most homologous mouse antibodies of solved crystal structure as templates to guide humanization of 3D6 [31-34]. The homology model of 3D6 Fv was built using pdb identifiers 1CR9 VL and 1OPG VH, respectively, (overall VL identity = 94% and VH identity = 72%) using QUANTA (Release dated 1999, Accelrys Software Inc., San Diego, CA, USA). The crystal structures of the selected modeling templates had been solved to high resolution (2 Å), and their primary sequence fulfilled key criteria to facilitate design of the humanized antibody based on the most homologous human VL (Kabat accession #019230) and VH (Kabat accession #045919) frameworks available at the time in public databases [35]. All antibody residue numbering in this report is according to Kabat [36], with the one exception as noted in the figure legend.

## Results

### Structure of 3D6 with Aβ peptide

**Figure 1:**
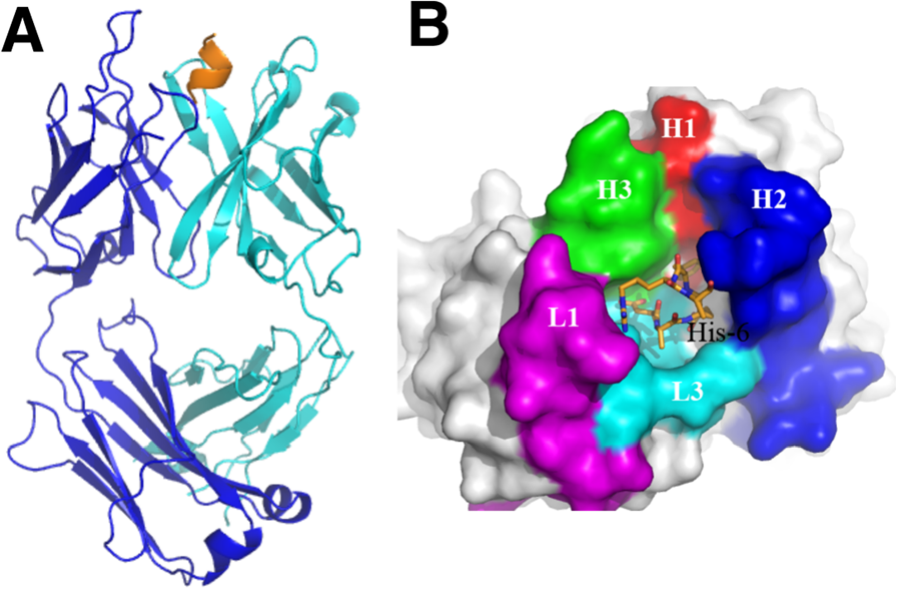
**Overview of the structure of 3D6 fab with Aβ1-7 peptide (pdb identifier 4ONF). A)** Side view showing alpha-carbon backbone traces of the molecules. Heavy chain is shown in cyan, light chain in blue and Aβ peptide in yellow/orange. **B)** 3D6 with Aβ1-7 view from above the molecule. Peptide is shown in stick representation with oxygens colored red, nitrogens in blue and carbons orange. Fab in surface representation colored white with the exception of CDRs contacting the peptide. CDR H1 is shown in red, H2 blue, H3 green, L1 magenta and L3 cyan. Only CDRs contacting the peptide are colored. CDRs, complementarity determining regions; pdb, protein data base.

**Figure 2:**
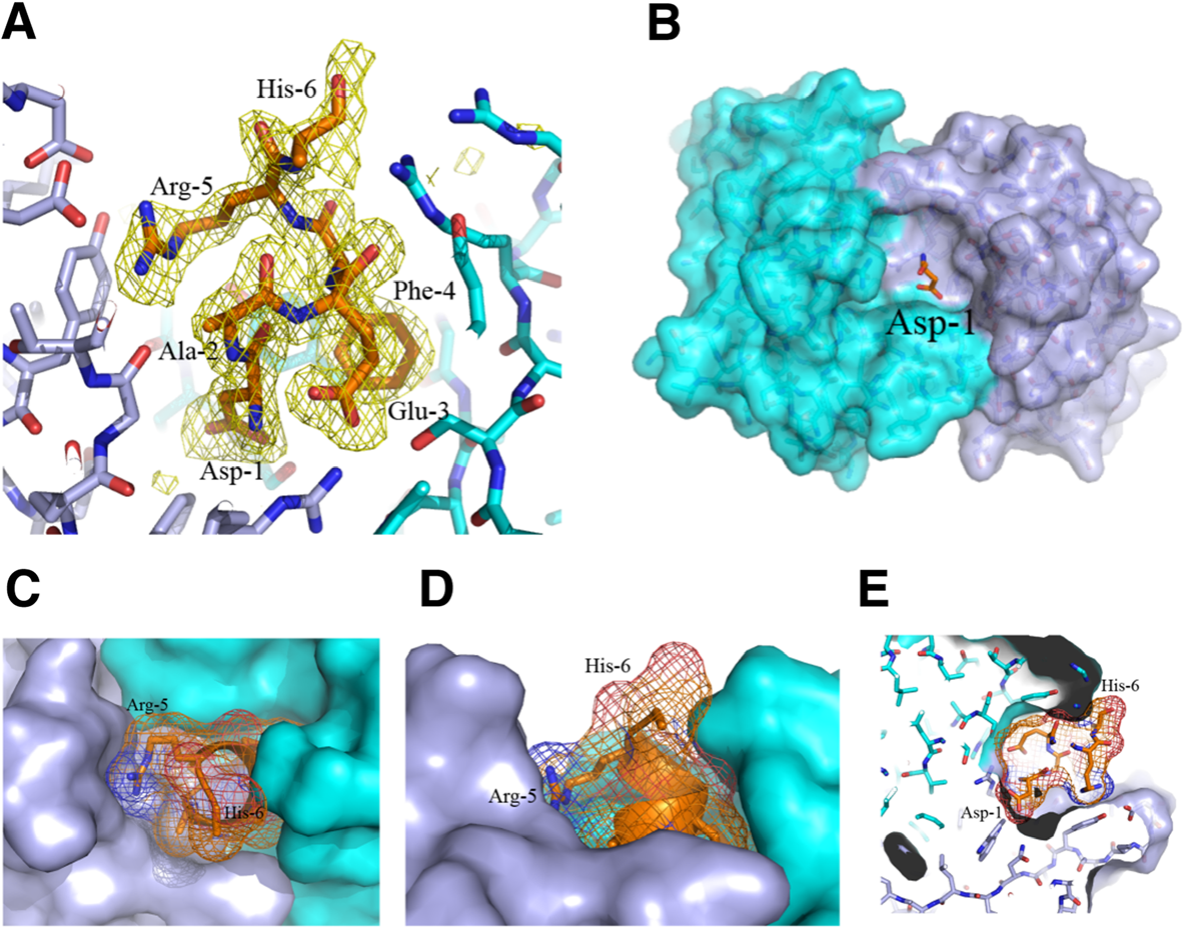
**Detailed view of Aβ 1–7 bound to 3D6. A)** 3.0 sigma electron density omit map (calculated while omitting the Aβ peptide from the model for phase calculations) is shown in orange. Protein and peptide are shown in stick representation with oxygens colored red, nitrogens in blue, carbons in orange for the peptides, light blue for the light chain and cyan for the heavy chain. **B, C, D, E)** The surface of the heavy chain is shown in cyan and light chain in light blue. Aβ peptide is shown in stick representation with oxygens colored red, nitrogens in blue and carbons in orange. In B only, peptide Asp-1 is shown. Asp-1 is buried inside a deep cave on the surface of the antibody. In C, D and E a mesh, representing the surface of the peptide, is shown to illustrate the actual volume of the peptide in the cave, note: the side chain of residue 6 is not visible beyond the Cβ atom of His-6. **C)** top view. **D)** side view. **E)** side view slice illustrating the depth of the cave.

**Figure 3:**
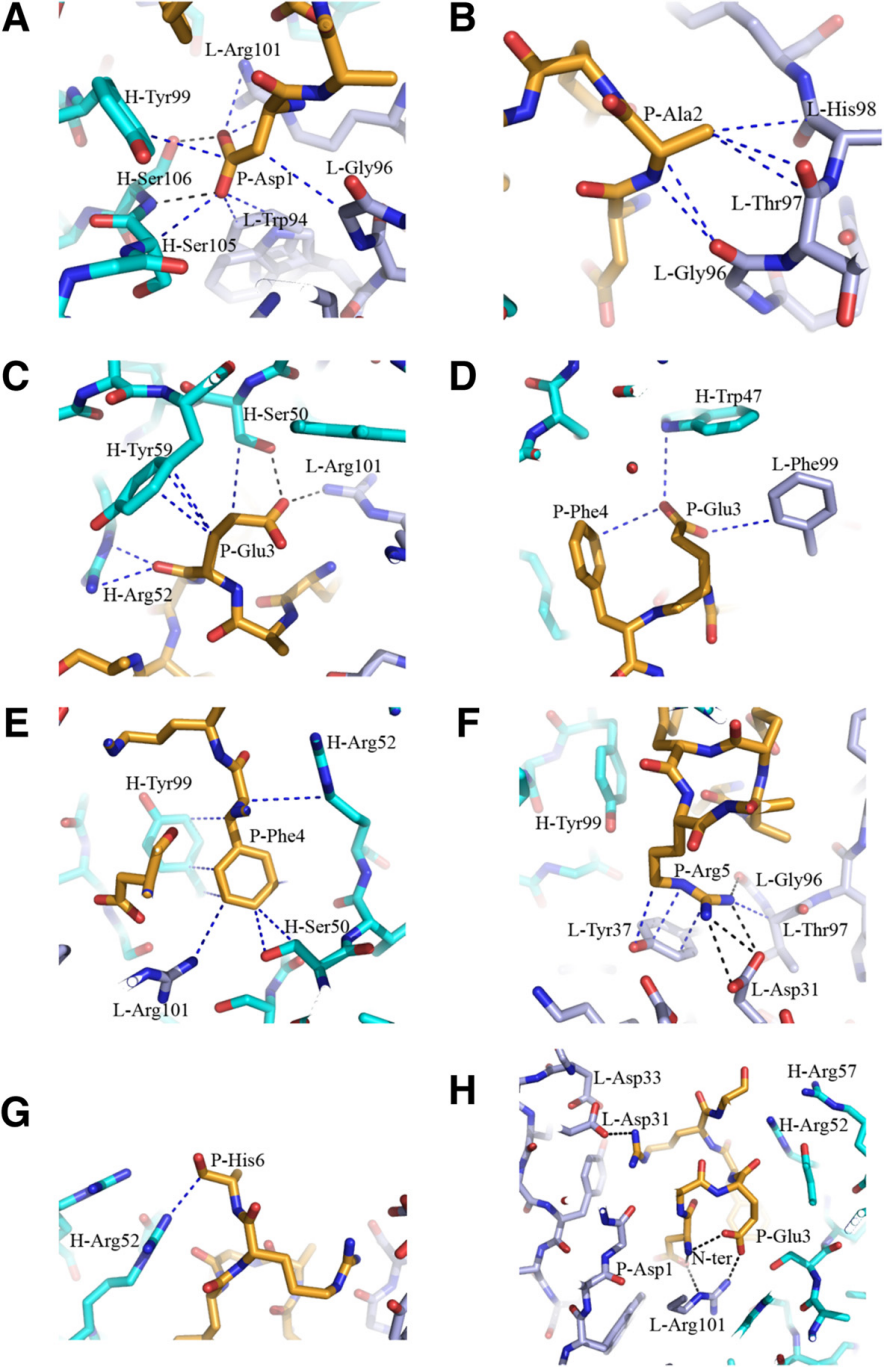
**Interaction of Aβ residues with 3D6.** For clarity, representative contacts per residue are shown. Protein and peptide are shown in stick representation with oxygens colored red, nitrogens in blue and carbons in orange for the peptides, light blue for the light chain and cyan for the heavy chain. Heavy chain and light chain residues are indicated by H and L prefix, respectively, Aβ peptide residues are indicated by the prefix P. The dotted lines indicate key contacts <4 Å. Interaction of Aβ residues 1,2,3,3,4,5,6 are shown in panels **A** to **G** respectively. The overall position of the peptide in the binding site is shown in panel H. Note: In panels **G** and **H** the side chain of residue 6 is not visible beyond the Cβ atom of His-6. The numbering of residues in Figure 3 matches the numbering in the x-ray coordinates file (4ONF) and differs from the Kabat numbering scheme employed in the rest of this manuscript.

Crystal structures of 3D6 Fab bound to Aβ peptides were solved to high resolution with x-ray crystallography, one with Aβ1-7, the second with Aβ1-40. The crystallographic data are summarized in Table 1. Due to the high degree of similarity between the two structures, observations from the 3D6:Aβ1-7 structure only are described in this report. The antigen binding site of 3D6 is in a cleft positioned in the interface between the heavy and light chain. The Aβ peptide lies in a deep fissure arranged by the complementarity determining regions (CDRs) H1, H2, H3, L1 and L3 of the Fab (Figure 1B) and adopts a 3_10_-helix conformation where the N-terminus of the peptide is buried in the interface between the VL and VH domains (Figures 1A and 2). Antigen contacting antibody residues are listed in Table 2, and illustrated in Figure 3. Aβ-Asp1 has close contacts with both the heavy and light chains, mainly interacting with CDR-H3 and CDR-L3. Aβ-Ala2 mostly interacts with the light chain comprising CDR-L3. Aβ-Glu3 mostly contacts the heavy chain CDR-H2 but also contacts light chain CDR-L3. Aβ-Phe4 mainly interacts with the heavy chain contacting CDR-H1, H2 and H3. Aβ-Arg5 is in close contact to the light chain CDR-L1 and CDR-L3. Aβ-His6 does not have well-defined electron density, but it appears to contact the heavy chain comprising CDR-H2 (Figure 3). The lack of electron density beyond the Cβ atom in Aβ-His6 indicates that the side chain of this residue is not bound by the antibody. Hence, the structure of the Fab:Aβ complex confirms the Aβ epitope recognized by 3D6 as previously mapped by ELISA [16].

**Table 2:** **Residues of the antibody contacting A**β **(that is, within 4 angstroms) in the structure of 3D6 with A**β**1-7**

**Aβ residue**	**Structure**	**VL contacts**	**VH contacts**	**Peptide contacts**
D1	1-7	W89 G91 R96	Y95H S100a S100b	A2 E3 F4
	4HIX	W89 G91 R96	Y95H S100a S100b	A2 E3 F4
A2	1-7	G91 T92 H93 F94		D1 E3 F4 R5 H6
	4HIX	G91 T92 H93 F94		D1 E3 F4 R5 H6
E3	1-7	F94 R96	W47 S50 R52 Y58	D1 A2 F4 R5
	4HIX	F94 R96	W47 S50 R52 Y58	D1 A2 F4 R5
F4	1-7	R96	G33 M34 S50 I51 R52 Y95	D1 A2 E3 R5
	4HIX	R96	G33 M34 S50 I51 R52 Y95	D1 A2 E3 R5
R5	1-7	D27d D28 Y32 G91 T92	Y95	A2 E3 F4 H6
	4HIX	D27d D28 Y32 G91 T92	R52 Y95	A2 E3 F4 H6

Antigen contacting antibody residues revealed by the structure of 4HIX [24] is shown for comparison. Residues are numbered according to [36].

**Figure 4:**
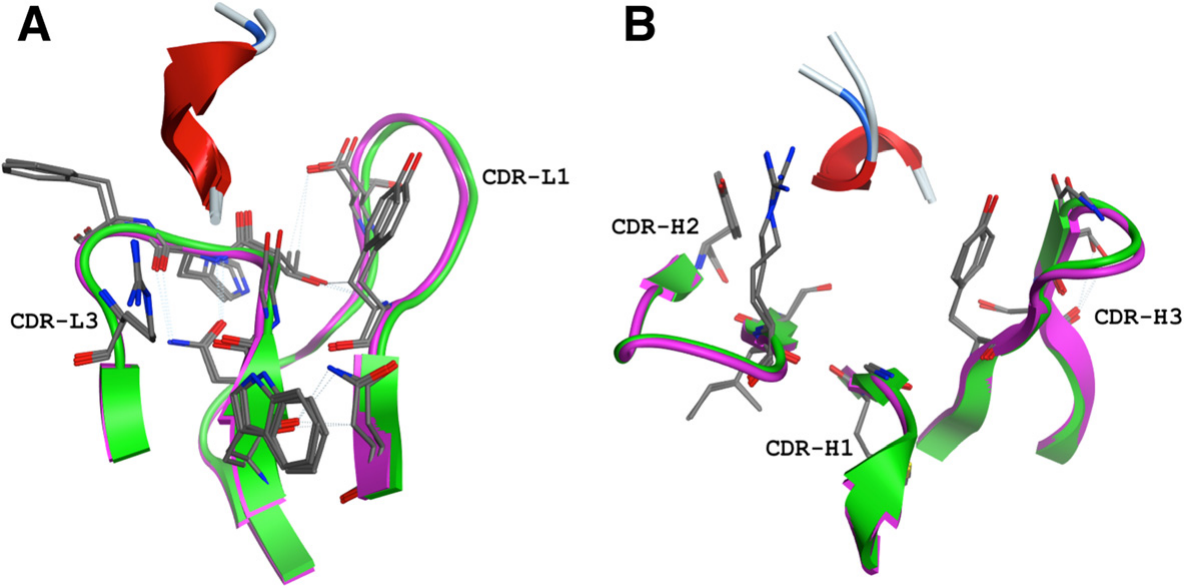
**Conservation of Aβ and CDR loop conformation comparing 3D6 and humanized 3D6v1. A)** Light chain CDR residues within 4 Å of antigen (some through water molecules which are not shown). 3D6 in purple, 4HIX structure [24] in green, antigen from both structures in red ribbon. **B)** Heavy chain CDR residues within 4 Å of antigen (some through water molecules which are not shown). 3D6 in purple, 4HIX structure in green, antigen from both structures in red ribbon. The dotted lines indicate hydrogen bonds. CDR, complementarity determining region.

The structure of 3D6 with Aβ1-7 reveals excellent agreement (alpha-carbon backbone root mean square deviation of atomic position (RMSD) = 0.56 Å) with the 4HIX structure [24]. The conservation of antigen contacting antibody residues, conformation of CDR loops, and conformation of antigen recognized are detailed below (Tables 2 and 3, Figure 4). Our structure of 3D6:Aβ contrasts with the structure of Aβ bound to gantenerumab [25], which was reported to recognize Aβ 2–9, with weak binding to residues 1 and 10, as well as the central epitope by pepspot analysis of overlapping decameric peptides of Aβ. Residues 1–11 of Aβ peptide in the gantenerumab structure were observed bound in an extended conformation, with the N-terminal to C-terminal orientation of Aβ reversed by 180° relative to other published structures, excluding 3D6 and 4HIX [23,26,37,38].

**Table 3:** Summary of mouse VL and VH framework residues retained in humanized 3D6/bapineuzumab based on predicted interactions (<4 Å) with critical distal intra-chain or inter-chain residues

**Mouse VL Fr residue retained in bapineuzumab**	**Interacting residues revealed from crystal structure of 3D6**	**Mouse VH Fr residue retained in bapineuzumab**	**Interacting residue revealed from crystal structure of 3D6**
V2	*Ser26:L1*, *Gln27:L1*	A49	*Ser35:H1*
	**His93:L3**, *Thr97:L3*		**Ser50:H2**, **Tyr58:H2***Tyr59:H2*
L36	**Trp89:L3**	V93	**Met34:H1**, *Ser35:H1*
	Leu45(H), Trp103(H)		**Tyr95:H3**, **Ser100B:H3***Tyr102:H3*
R46	Trp35(L), Leu36(L) *Asp55:L2*, Val58(L)	R94	*Tyr32:H1*, **Met34:H1**
			**Tyr95:H3**, *Asp96:H3***Ser100B:H3**, *Asp101:H3 Tyr102:H3*
	*Ser99:H3*, **Ser100a:H3 *Ser100b:H3****, Asp101:H3*		

The residues were retained based on model predictions. The crystal structure of 3D6 confirmed interactions <4 Å between the retained mouse residues and the residues listed above. CDR residues revealed in the structure of 3D6 to directly contact Aβ peptide antigen (from Table 2) are shown as boldface, italicized and boldface font indicates CDR residue that makes an α-carbon backbone contact with the retained mouse residue. CDR residues not contacting antigen are indicated by italics. L1, L2 and L3 indicate light chain CDR1, CDR2, and CDR3 residues respectively, and H1, H2, and H3 indicate heavy chain CDR1, CDR2 and CDR3 residues. Framework (Fr) residues are indicated as unformatted text followed by (H) and (L) to indicate heavy or light chain origin, respectively. CDR, complementarity determining region.

Several interactions between the peptide and the antibody involve bridging water molecules (also observed in the 4HIX crystal structure [24]. Water molecules are present not only on the surface of the antibody but also inside the deep fissure at the antibody binding site, and interact with residues from the N-terminus of the peptide that are positioned at the bottom of the elongated site. Examples are shown in Additional file 2: Figure S1. As also noted by Miles *et al*. [24], a helical conformation of Aβ has been revealed via nuclear magntic resonance (NMR) spectroscopy [39,40]. Whereas the structure of the amyloidogenic core of Aβ peptide has been extensively characterized (see for example, [41-43]), it is well recognized that the amino-terminus of Aβ does not adopt a single conformation in solution. Coupled with the solution NMR data, our results are consistent with the notion that the amino terminus dynamically samples a helical conformation, and it is this subpopulation of Aβ that is recognized by 3D6. A hypothetical fit of the NMR structure of Aβ with our structure is illustrated in Additional file 3: Figure S2. Given the absence of electron density after residue 5 in the Aβ1-40 complex, it is likely that upon binding the amino-terminal epitope, the antibody can interact with different conformations adopted by the Aβ peptide past residue 5.

### Comparison of 3D6 and 12A11 binding to Aβ peptide

**Figure 5:**
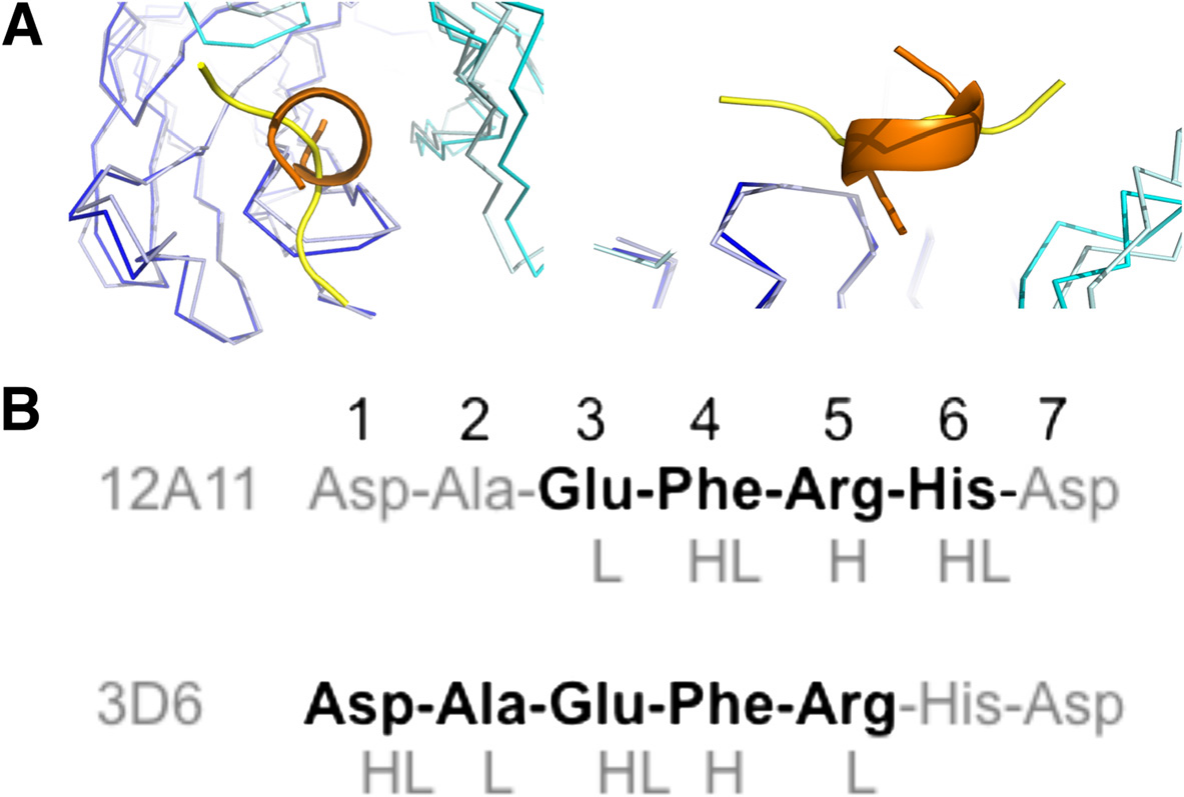
**Comparison of 3D6 (pdb identifier 4ONF) and 12A11 (pdb identifier 3IFN) binding to Aβ. A)** Superposition of the α-carbon backbone of 3D6 and 12A11 [23] illustrating different conformations of antigen captured by antibody as revealed from the crystal structures. For 3D6, the heavy chain is shown in cyan, light chain in blue and Aβ peptide in orange; for 12A11, the heavy chain is shown in light-cyan, light chain in light-blue and Aβ peptide in yellow. Left – top view. Right – side view. **B)** Primary structure from N-terminus Aβ, illustrating the epitope recognized by 12A11 versus 3D6 in boldface. CDRs contacting antigen at each residue of the minimal epitope are indicated below the primary structure (L-light chain CDR, H-heavy chain CDR). CDRs, complementarity determining regions; pdb, protein data base.

Both 3D6 and 12A11 recognize different but overlapping N-terminal epitopes in the Aβ peptide, with an overlap of three residues in the central region of the shared epitope (Figure 5B). While 12A11 captures an extended conformation of the peptide that is highly conserved among antibodies binding this epitope [25,26,37,38], 3D6 binds Aβ in a 3_10_-helix conformation (Figure 5A). The CDR sequences of 12A11 and 3D6 do not have high similarity, nor do the interactions of the CDRs with the peptide. Although the antigen-binding site in both cases is in the interface between the heavy and light chain, the antigen binding cleft in 3D6 is very deep, where the N-terminus of the peptide is positioned at the bottom of the fissure. In contrast, the antigen-binding groove of 12A11 is shallow, with only the side chains of the peptide buried in the crevice [23]. In 3D6 the peptide lies in a deep fissure as described above, while in 12A11 the peptide adopts an extended conformation along the groove that is composed by CDRs H2, H3 L1 and L3 of the Fab. Because the variation in the binding conformation is large, and the CDRs interact with different (but overlapping) epitopes in the peptide (Figure 5), a direct comparison of CDR sequences is not relevant.

### Structure of 3D6 Fab:Aβ complex supports design considerations in humanization to bapineuzumab

Humanization of 3D6 was effected by a combination of CDR grafting of mouse residues from 3D6 VL and VH into human frameworks, and homology model guided retention of select mouse framework residues in the initial humanized version of the monoclonal antibody (mAb) [31,32,35,44,45]. Superposition of the model with the solved crystal structure of 3D6 gives an RMSD 0.74 Å for the VL domain, and 1.93 Å for VH (primarily attributable to expected deviation in CDR-H3). The overall RMSD for Fv is 2.35 Å, arising from a difference in the orientation of VL and VH with respect to each other. Two versions of humanized 3D6 were expressed and tested (detailed below), and both versions were demonstrated to retain all desired *in vitro* and *in vivo* properties of the parent murine mAb [46], for example, retention of affinity for antigen, stimulation of plaque phagocytosis by microglia in a frozen section *ex*-*vivo* assay, ability to recognize β-amyloid in plaque by histology of PDAPP and AD brain in frozen section, brain localization in PDAPP and epitope specificity.

**Figure 6:**
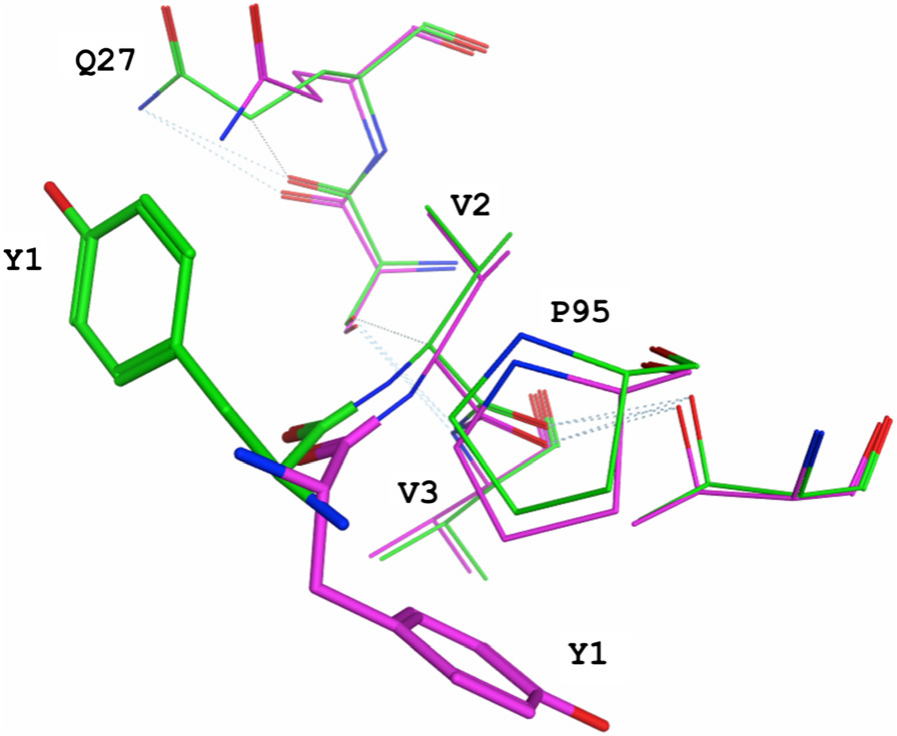
**Comparison of the different conformations for Y1 at the N-terminus of the light chain.** Structure of 3D6 shown in purple and 4HIX [24] structure shown in green. The dotted lines indicate hydrogen bonds.

The structural contributions of several residues mutated to human, or retained as mouse, in the two versions of humanized 3D6 are of interest. The light chain variable region of 3D6 contains a Tyr at its amino terminus, a rare residue at this position in human VL sequences. The crystal structure of 3D6 reveals that the tyrosine residue is placed between CDRs L1 and L3. It interacts with solvent and is mobile, evidenced by its two very different conformations in 3D6 and 4HIX [24], illustrated in Figure 6. In 3D6 the Y1 aromatic ring is closer to P95 of CDR-L3, whereas in 4HIX it is closer to Q27 of CDR-L1. It should be noted that while the structure reported by Miles *et al*. [24] has Tyr at position 1 of the light chain, this residue was determined to be dispensable for retention of affinity in version 2 of humanized 3D6 (Table 4, compare h3D6v1 versus h3D6v2). Consequently, a mutation of Y1D (the more commonly observed residue at this position in human VL domains) was carried out during humanization of 3D6 to bapineuzumab in order to minimize potential immunogenicity.

**Table 4:** Binding kinetics of murine, recombinant chimeric, and two versions of humanized 3D6 to Aβ1-10 peptide

**Antibody**	**ka1 (1/Ms)**	**kd1(1/s)**	**K**_**D **_**(nM)**	**SD**	**n**
Mu 3D6	3.61E + 05	4.12E-04	1.2	0.22	3
Chi 3D6	3.43E + 05	5.89E-04	1.8	0.73	5
Hu 3D6 v1	1.89E + 05	3.99E-04	2.1	0.07	2
Hu 3D6 v2	1.50E + 05	4.23E-04	2.8	0.54	4

"Mu" denotes Murine, "Chi" denotes chimeric, "Hu" denotes humanized. Version two of humanized 3D6 was selected for clinical development (see text). Specific residues in v2 of humanized 3D6 (bapineuzumab) were Y1D, *V2*, *L36*, and *R46* in VL, and N40A, D42G, *A49, V93*, A75S, S78T, L93V and *R94* in VH, where italicized residues denote mouse Fr residues retained during humanization. In the table headings, ka1 denotes on rate, kd1 denotes off-rate, K_D_ indicates affinity, SD denotes standard deviation of K_D_ measure, and n indicates the number of independent replicates in the measure of affinity for each version of the antibody tested.

The murine VL framework residues Val2, Leu36 and Arg46 were retained in bapineuzumab based on the model, which predicted interactions of Val2 with residues in CDR L1 and L3 (see Additional file 4: Figure S3). Leu36 was retained based on its predicted role for inter-chain packing, while Arg46 was predicted to be involved in interactions with H3, in addition to multiple contacts with non-contiguous VL Fr residues. These predictions were confirmed in the crystal structure of 3D6 as summarized in Table 3, and detailed in Additional file 5 ‘Structural observations in 3D6 regarding mouse VL Fr residues retained during humanization, confirming predictions based on homology model*’*.

**Figure 7:**
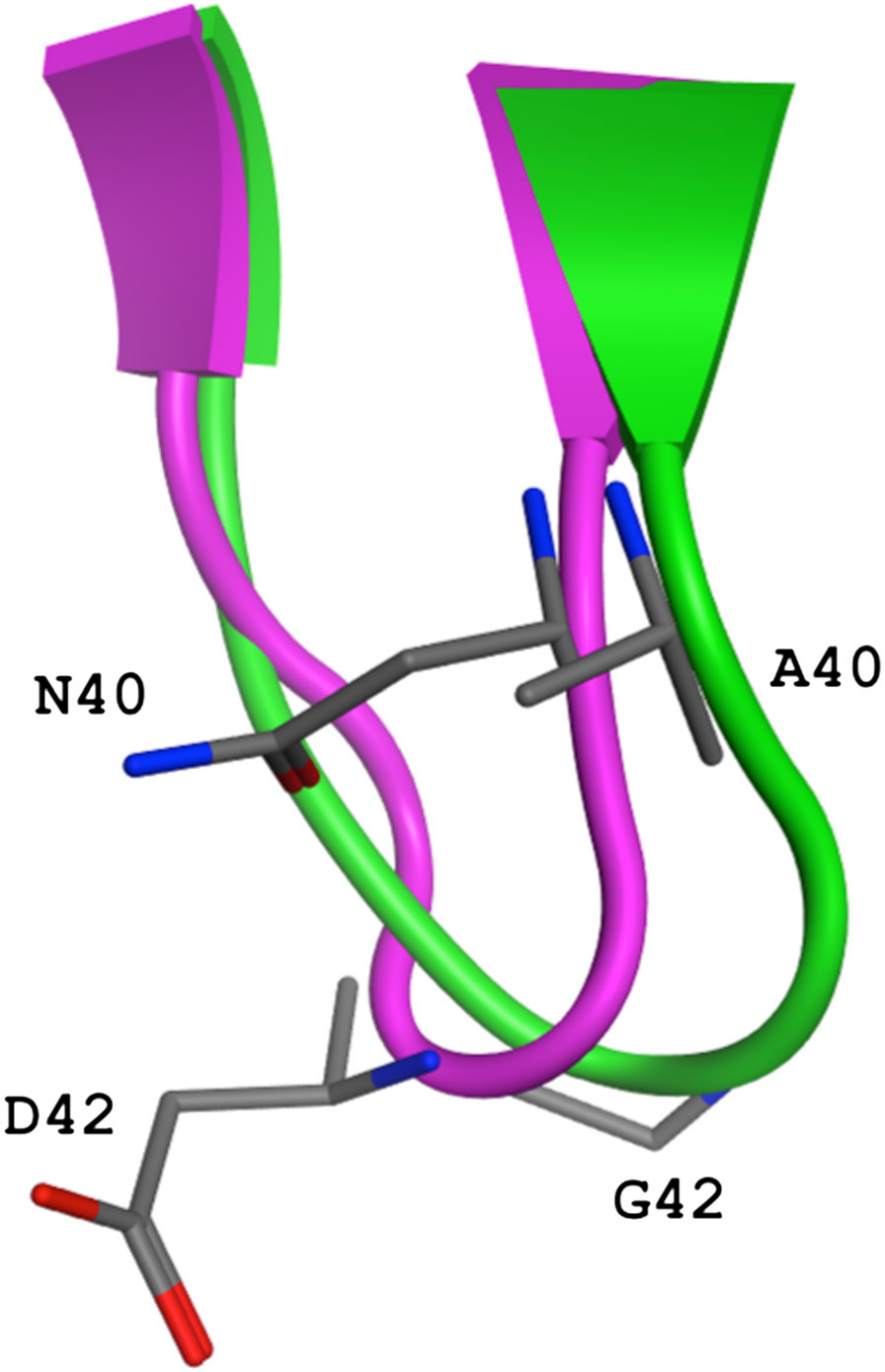
**Conformational change of a beta turn in the alpha-carbon backbone effected by humanization of 3D6.** Ribbon depiction of the change in turn type with mutations N40A and D42G. 3D6 in purple (type I turn) and 4HIX [24] structure in green (type II turn) with stick representation for the mutated side-chains.

The design of humanized 3D6 VHv1 involved replacement of two rare mouse residues (Asn40, Asp42) in FR2 with the more frequent and preferred human residues at those positions (Ala40, Gly42). Examination of the structure reveals Asn40 and Asp42 are situated on a Type I β-turn between β-strands 3 and 4 of the VH. Asn40 forms a hydrogen bond with the main-chain nitrogen of Arg44, and Asp42 interacts with the Arg44 sidechain via a water molecule. Replacement of these surface exposed rare mouse amino acids with the human framework counterparts was considered prudent from the perspective of eliminating potential immunogenicity [32,47]. Interestingly, N40A and D42G mutations, coupled with changing mouse Fr residue S41 to human Fr residue P41, resulted in a change of conformation from a Type I β-turn to a Type II β-turn in this region of the molecule (Figure 7). It is not clear if this change in conformation is attributable to the S41P alone, or the triple mutation in combination. Nevertheless, it did not alter the affinity (Table 4), or other activities of the humanized antibody summarized below (see also [46]).

The three mouse VH framework residues retained in humanized 3D6 VHv1 (Ala49, Val93, and Arg94) are all revealed by the crystal structure of 3D6 to mediate contacts with CDR residues, contributing to preservation of CDR conformation, and by extension, contributing to antibody:antigen contacts (Table 3, and Additional file 5 ‘Structural observations in 3D6 regarding mouse VL Fr residues retained during humanization, confirming predictions based on homology model’).

Finally, three human VH Fr residues in v1 of humanized 3D6 were converted to germline A75S, S78T and L93V based on the sequence of the closest human germline VH region (V3-23), leading to version two of humanized 3D6 (bapineuzumab). The germline changes were incorporated in bapineuzumab to minimize potential Fr changes in the human VH (KABID 045919) arising from somatic hypermutation during affinity maturation. The crystal structure of 3D6 reveals that the methyl side chain of Ala75 is exposed to solvent on a β-turn. Conversion to germline of human Ser78 to Thr actually restores the residue at this position in 3D6, and preserves intra-chain cross β-strand interactions between T78 and residues on β-strand 1 of VH. The Leu93Val germline change is a conservative mutation that would preserve local interactions of the original Fr Leu residue. These germline changes in bapineuzumab VH were not incorporated into the molecule crystallized by Miles *et al*. [24] (pdb identifier 4HIX). Version two of humanized 3D6 was advanced for clinical development as bapineuzumab. The integrity of the sequence of bapineuzumab was confirmed by mass spectroscopy, and amino acid analysis (AAA) (data not shown, reported in the chemistry, manufacture, and controls (CM and C) section of the investigative new drug (IND) application submitted to the US Food and Drug Administration (FDA) in support of the clinical development of bapineuzumab).

While the contribution of individual light and heavy chain framework residues described in this section to antibody activity (particularly affinity) must await confirmation via systematic site directed mutation analyses in future studies, the activity of bapineuzumab was compared with 3D6 *in vitro* as well as *in vivo* in a number of different assays [46]. Binding kinetics and affinity (a primary criteria in antibody humanization) measurements comparing 3D6 with bapineuzumab were carried out by surface plasmon resonance. The data comparing 3D6 versus two versions of humanized 3D6 binding to an Aβ1-10 peptide antigen, summarized in Table 4, revealed that both versions of humanized 3D6 retained binding affinity for antigen within the target 2-3X of parent mouse antibody, although this relative difference in affinity measures appears insignificant when referenced to the chimeric antibody. (See Additional file 6: Figure S4 for representative surface plasmon resonance binding curves underlying the data in Table 4.) The conformation of CDR loops, conformation of antigen binding residues, as well as the conformation of bound antigen, reveals excellent conservation of structure between 3D6 and 4HIX (Figure 4), supporting the design algorithm employed in the humanization of 3D6 to bapineuzumab.

## Discussion

Multiple groups have reported the structural features of antigen recognition and antigen conformation from anti-Aβ antibodies targeting an amino-terminal epitope of the peptide [23-26,37,38]. The neo-epitope specificity of 3D6 combined with conformation of the bound epitope, elucidated here at 2 Å resolution and in the structure 4HIX [24], is clearly unique among the antibodies studied to date, as well as among clinical candidates under current investigation. Gantenerumab [25] binds Aβ in a manner that overlaps the epitope recognized by 3D6 and offers an interesting comparison. Although gantenerumab recognizes two discontinuous epitopes on Aβ by pepspot analysis, the crystal structure revealed binding to Aβ residues 1–11. The reported crystal structure for gantenerumab crystals soaked with Aβ1-11 is not publicly available, limiting a detailed comparison with our structure of 3D6 with Aβ. However, in contrast with the 3_10_ helical conformation captured by 3D6, Aβ is described as binding gantenerumab in an extended conformation with antibody residues from H1, H2, H3 and L3 contributing sites of contact with antigen. This further contrasts with our observations with antibody 3D6 CDRs, where CDRs H1, H2, H3, L1 and L3 contribute sites of contact with Aβ (Table 2). Thus, available evidence suggests that the conformation of Aβ recognized by gantenerumab is distinct from 3D6, and apart from a reversed amino to carboxy terminal orientation, is more similar to WO2 [38], 12A11 [23] and PFA1 [37]. Hence, the amino-terminal conformation of Aβ recognized by 3D6, and most likely also bapineuzumab as evidenced by 4HIX, comprises a third conformation adopted by this non-amyloidogenic and relatively unstructured domain of the peptide.

In conjunction with the wealth of biophysical studies of the structure of the core amyloidogenic region of Aβ in fibrils (see for example, [41-43]), the combined data sets from antibody crystallization studies provide a structural snapshot of the amino-terminal conformations adopted by one of the two important pathologic determinants of AD. Based on the distinct conformations of Aβ recognized by 3D6 (helical) versus 12A11, gantenerumab, WO2, PFA1 (extended), it is reasonable to speculate that 12A11 and 3D6 target different amino-terminal conformations of Aβ peptide presented in its multiple states. Consequently, one could envision a scenario whereby antibody binding to the amino terminal epitope of Aβ alone could skew the equilibrium distribution of total Aβ species (soluble monomer to amyloid plaque deposited forms) toward the antibody bound state, and thereby potentially affect the overall steady-state distribution of the different forms in which Aβ may exist at any given point in time.

Although the structure of 3D6:Aβ reported here is in very close agreement with its humanized version 4HIX [24], the discrepancy between our reports in the affinity of humanized 3D6 v1 (equivalent to 4HIX) merits comment. We observed low nanomolar affinity for Aβ with both versions of humanized 3D6 and a 2-3X loss of affinity between murine or chimeric 3D6 and humanized (Table 4). Miles *et al*. reported an affinity of 76 nM for Aβ1-40, 150 nM for Aβ1-28, and low μM for Aβ1-7, from their synthetic Fab of humanized 3D6 (equivalent to our version 1)*.* These values reflected a 15 to 30 fold loss of affinity in the humanized mAb when compared to a 5 nM affinity of 3D6 for Aβ1-40 reported by DeMattos *et al*. [48]. Our measure of the affinity of 3D6 for Aβ1-10 is in closer agreement with that reported by DeMattos *et al*. The discrepancy between affinity measurements reported here (and by DeMattos *et al*.), versus the affinity reported by Miles *et al*. can be attributed primarily to the use of whole antibody (this report and DeMattos *et al*.) versus Fab (Miles *et al*.). A secondary contributor to our discrepant affinity measures may be attributable to differences in methodology for affinity determination (surface plasmon resonance in our report and microscale thermophoresis by Miles *et al*. [24]). The structural details for antigen recognition by antibody provide insight toward modulating affinity via site directed mutagenesis.

Antibody humanization provided the first approach to solving the limiting immunogenicity of mouse antibodies in man [32,44,47,49-51]. Antibody engineering technology has been continuously evolving over that time period, from pioneering work with phage display [52-56] and xeno-mouse based approaches [57-59] to more esoteric recombinant engineering of xenogenic species derived antibodies and yeast display based approaches (for reviews, see [60-62]). The excellent agreement we report between the structures of 3D6:Aβ and that of 4HIX, as summarized in Table 2 and Figure 4, attests to the robustness and continued viability of antibody humanization as a rapid and efficient technology for lead optimization of biological drug candidates. The approaching expiry of the exclusivity period of archetypal humanization patents [63-66] provides an illustrative (but not comprehensive) rationale for the continued value of antibody humanization as an economical platform for drug development going forward.

A bi-specific [67,68] or dual variable domain [69] antibody combining recognition of the two conformations of Aβ revealed from crystal structure studies, that is, extended conformation on one arm and helical conformation on the other arm, would offer intriguing dual activity that could be particularly well suited for disaggregation of β-amyloid plaque independent of effector cell engagement. Recent progress with immunotherapy targeting Tau in preclinical models is revealing preferred targets for antibody binding [70]. Structural investigation of the conformation of tau recognized by efficacious anti-Tau antibodies will be informative regarding the mechanisms underlying intercellular transmission of these pathological entities, and furthermore, enable design of bi-specific antibodies capable of recognizing and neutralizing both tau and Aβ.

## Conclusions

Disease modifying therapy of AD remains a significant unmet medical need [71-74]. The disease modifying effects of Aβ-targeted immunotherapy [4] have been widely reproduced in preclinical models. Although the initial clinical trials with bapineuzumab failed to achieve the clinical end-points at the disease stage tested [11]*,* the continued refinement of Aβ targeted immunotherapy for AD has resulted in numerous follow-on passive and active immunotherapy approaches currently undergoing clinical investigation. The efforts underway target either different epitopes of Aβ, (for example, Solaneuzumab [75] and Gantenerumab [15]), different species of Aβ (for example, oligomer specific antibodies discovered via a range of technologies [76-78] versus plaque specific [48]), or engage different mechanisms to effect clearance or neutralization of Aβ mediated pathologies, for example, effector function (Crenezumab [12] versus bapineuzumab [3] and gantenerumab [15]). The report by Lu *et al*. demonstrating conformational variation of β-amyloid fibril structure in AD patients [79] suggests utility as well as limitations of approaches targeting a specific conformation. As we have reported, 3D6 shows the ability to target and neutralize multiple species of Aβ [5], in an Fc receptor dependent as well as independent manner. Current evidence based on pre-clinical studies suggests that attenuated effector activity may be preferable in an immunotherapeutic targeting Aβ [20]. Hence, a second generation anti-Aβ therapeutic with attenuated effector function, as embodied in crenezumab and AAB-003 (containing humanized 3D6 variable regions), or a bi-specific antibody targeting different Aβ amino-terminal conformations, or both Aβ and Tau (as discussed earlier), which targets all forms of pathogen to neutralize, or promote, its clearance, may prove to be efficacious when applied in a disease stage appropriate manner [71,74].

## Abbreviations

3D6Aβ1-40: Fab 3D6 + Aβ residues 1–40; 3D6Aβ1-7: Fab 3D6 + Aβ residues 1–7; AAA: amino acid analysis; AD: Alzheimer’s disease; APP: amyloid precursor protein; Aβ: amyloid beta peptide; BACE: beta-site APP cleavage enzyme; CDR: complementarity determining region; CM and C: chemistry, manufacture, and controls; ELISA: enzyme linked immunosorbent assay; Fab: fragment antigen binding, comprising variable and first constant regions of heavy and light chains of an antibody; Fc: fragment constant, that is, constant region of antibody comprising the hinge, second and third constant regions of immunoglobulin heavy chain; Fr: framework; Fv: fragment variable, that is, just the VL and VH fragments of an antibody; H1: CDR1 in VH; H2: CDR2 in VH; H3: CDR3 in VH; IND: investigative new drug; L1: CDR1 in VL; L2: CDR2 in VL; L3: CDR3 in VL; PDAPP: PDGF promoter driven APP transgenic mouse model of AD; pdb: protein data base; PDGF: platelet-derived growth factor; RMSD: root mean square deviation of atomic position; TLS: Translation Libration Screw; v: version; VH: variable region, heavy chain; VL: variable region, light chain.

## Competing interests

All co-authors with the exception of Drs. William Weis and Hadar Feinberg were employees of and shareholders in either Elan Pharmaceuticals or Wyeth Inc., during the time this work was performed. Jose Saldanha was a consultant to Elan Pharmaceuticals, Inc.. Drs. Schenk, Basi, and Saldanha are co-inventors on patents related to Aβ immunotherapy.

## Authors’ contributions

HF conducted crystallography, data analysis, generated figures, and contributed to writing of the manuscript. JS provided the design of humanized 3D6, conducted data analysis comparing murine and humanized structures, generated figures, and contributed to writing of the manuscript. LD purified recombinant Fab for x-ray crystallization. AG conducted scale-up expression recombinant Fab. AW conducted biacore studies, performed data analysis and contributed to writing of the manuscript. GV designed and supervised kinetic studies, reviewed the biacore data, and contributed to writing of the manuscript. WIW supervised x-ray crystallography and audited x-ray data, partnered in establishing collaboration with Elan, and contributed to writing of the manuscript. DS established the collaboration with the Stanford group, and contributed to writing of the manuscript. GSB conceived of the study, supervised the molecular biology activities involving protein expression, and antibody humanization, and contributed to writing the manuscript. All authors read and approved the final manuscript.

## Supplementary Material

Additional file 1: Table S1 Crystallization conditions. Conditions for crystallization of 3D6 Fab with Aβ1-6 peptide, and Aβ1-40 peptide, are listed. Click here for file

Additional file 2: Figure S1 A pdf file. Some examples for 3D6 water mediated interaction with Aβ1-7. Protein and peptide are shown in stick representation with oxygens colored red, nitrogens in blue and carbons in orange for the peptides, light blue for the light chain and cyan for the heavy chain. The dotted lines indicate key contacts <4 Å. Click here for file

Additional file 3: Figure S2 A pdf file. Model for Aβ binding by 3D6. The model for the peptide was prepared by: 1. Superpositioning residues 1–5 of the NMR structure of Aβ1-28, pdb id 1AMB (first structure in the NMR pdb file) on 1–5 of 3D6. 2. Superpositioning of the longer Aβ1-42 NMR structure (the first structure in the pdb file, pdb id 1IYT) on 1AMB (residues 18–25) The final model for the peptide contains: residues 1–5 from the peptide bound in the 3D6 + Aβ1-7 peptide structure **(A)**, residues 6–26 from 1AMB [40]**(B)** and residues 27–42 from 1IYT [39]**(C)**. The final model was regularized in the section where two models connect (±2 residues, residues 3–7 and 24–28) to bring the bonds in the connection to a reasonable range **(D)**. In the final model **(E, F)** some clashes are observed in the region of H-Arg-52 and H-Arg-57, residues that are part of CDR-H2, and peptide residue Aβ-Asp7. A possible resolution for the observed clashes could be envisioned if the loop comprising CDR-H2, which contains many Gly residues, were to adopt a slightly different conformation. Alternately, if the rotamers for H-Arg-52, H-Arg-57, H-Tyr-59 and Aβ-Asp-7 were to be different, the modeled protein would be able to accommodate the conformation of the peptide illustrated and overcome the clashes. Click here for file

Additional file 4: Figure S3 A pdf file. Interaction of framework residue with CDRs. VL residue V2 (thick purple stick in center) which was retained in bapineuzumab based on predicted interaction with residues in CDR-L1 and L3. L1 shown in dark blue and L3 in light blue. Hydrogen bonds are shown as dotted lines. Click here for file

Additional file 5 ‘Structural observations in 3D6 regarding mouse VL Fr residues retained during humanization, confirming predictions based on homology model.’ Click here for file

Additional file 6: Figure S4 A pdf file. Surface plasmon resonance binding profiles of murine-3D6 IgG2b (red), chimeric-3D6 IgG1 (blue) and humanized-3D6 v2 (green) are shown on 5.0 RU of biotinylated-Aβ1-10 peptide immobilized on streptavidin coated chip. Click here for file
