# Supplementary material for: Crystal structure reveals conservation of amyloid-β conformation recognized by 3D6 following humanization to bapineuzumab
Source: Alzheimers Res Ther. 2014 Jun 2;6(3):31. doi: 10.1186/alzrt261 (PMC4095729; doi:10.1186/alzrt261)
Supplement: Additional file 1: Table S1 — Crystallization conditions. Conditions for crystallization of 3D6 Fab with Aβ1-6 peptide, and Aβ1-40 peptide, are listed. [file alzrt261-S1.docx]

**Additional Table 1: Crystallization conditions.**

Conditions for crystallization of 3D6 Fab with Aβ1-6 peptide, and Aβ1-40 peptide, are listed.

|  | **3D6Aβ1-7** | **3D6Aβ1-40** |
| --- | --- | --- |
| **Protein concentration (mg/ml)**  **In 10 mM Hepes, pH 7.5**  **75 mM NaCl** | 7.1 | 14.3 |
| **Protein:peptide molar ratio** | 1:2* | 1:1.8* |
| **Crystallization method**  **Protein:reservoir ratio (μl:μl)** | hanging drop  1:2 | sitting drop  0.25:0.25 |
| **Reservoir composition** | 30% PEG400  0.1MTris,  pH 9.0 | 2.5M NaCl  0.1M Imidazole,  pH 8.0  0.2M Zn(CH_3_COO)_2_ |
| **Freezing condition** | Reservoir | Reservoir + 20% Glycerol |

- Aβ stock solution (6.5mM) was prepared in DMSO.
