# Supplementary material for: Crystal structure reveals conservation of amyloid-β conformation recognized by 3D6 following humanization to bapineuzumab
Source: Alzheimers Res Ther. 2014 Jun 2;6(3):31. doi: 10.1186/alzrt261 (PMC4095729; doi:10.1186/alzrt261)

Additional Figure 1.

Some examples for 3D6 water mediated interaction with Aβ1-7. Protein and peptide are shown in stick representation with oxygens colored red, nitrogens in blue and carbons in orange for the peptides, light blue for the light chain and cyan for the heavy chain. The dotted lines indicate key contacts < 4 Å.

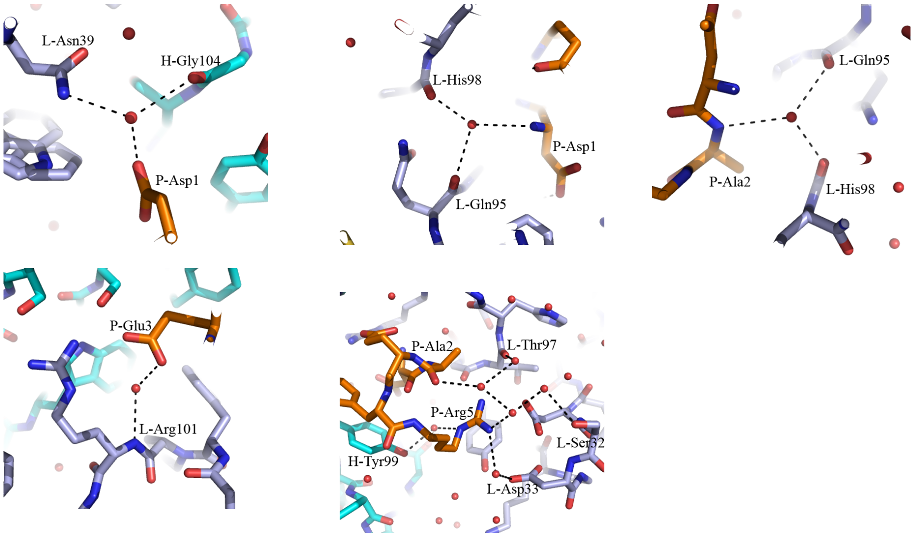

Supplement: Additional file 2: Figure S1 — A pdf file. Some examples for 3D6 water mediated interaction with Aβ1-7. Protein and peptide are shown in stick representation with oxygens colored red, nitrogens in blue and carbons in orange for the peptides, light blue for the light chain and cyan for the heavy chain. The dotted lines indicate key contacts <4 Å. [file alzrt261-S2.pdf]
