# Supplementary material for: Crystal structure reveals conservation of amyloid-β conformation recognized by 3D6 following humanization to bapineuzumab
Source: Alzheimers Res Ther. 2014 Jun 2;6(3):31. doi: 10.1186/alzrt261 (PMC4095729; doi:10.1186/alzrt261)

Additional Figure 2: Model for Aβ binding by 3D6. The model for the peptide was prepared by:

1. Superpositioning residues 1-5 of the NMR structure of Aβ1-28, pdb id 1AMB (first structure in the NMR pdb file) on 1-5 of 3D6.

2. Superpositioning of the longer Aβ1-42 NMR structure (the first structure in the pdb file, pdb id 1IYT) on 1AMB (residues 18-25)

The final model for the peptide contains: residues 1-5 from the peptide bound in the3D6 + Aβ1-7 peptide structure (A), residues 6-26 from 1AMB [40] (B) and residues 27-42 from 1IYT [39] (C). The final model was regularized in the section where two models connect (±2 residues, residues 3-7 and 24-28) to bring the bonds in the connection to a reasonable range (D).

In the final model (E, F) some clashes are observed in the region of H-Arg-52 and H-Arg-57, residues that are part of CDR-H2, and peptide residue Aβ-Asp7. A possible resolution for the observed clashes could be envisioned if the loop comprising CDR-H2, which contains many Gly residues, were to adopt a slightly different conformation. Alternately, if the rotamers for H-Arg-52, H-Arg-57, H-Tyr-59 and Aβ-Asp-7 were to be different, the modeled protein would be able to accommodate the conformation of the peptide illustrated and overcome the clashes.

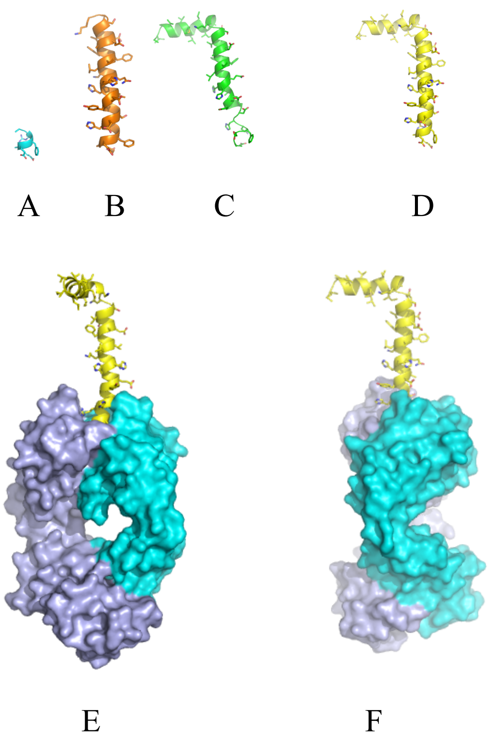

Supplement: Additional file 3: Figure S2 — A pdf file. Model for Aβ binding by 3D6. The model for the peptide was prepared by: 1. Superpositioning residues 1–5 of the NMR structure of Aβ1-28, pdb id 1AMB (first structure in the NMR pdb file) on 1–5 of 3D6. 2. Superpositioning of the longer Aβ1-42 NMR structure (the first structure in the pdb file, pdb id 1IYT) on 1AMB (residues 18–25) The final model for the peptide contains: residues 1–5 from the peptide bound in the 3D6 + Aβ1-7 peptide structure (A), residues 6–26 from 1AMB [40](B) and residues 27–42 from 1IYT [39](C). The final model was regularized in the section where two models connect (±2 residues, residues 3–7 and 24–28) to bring the bonds in the connection to a reasonable range (D). In the final model (E, F) some clashes are observed in the region of H-Arg-52 and H-Arg-57, residues that are part of CDR-H2, and peptide residue Aβ-Asp7. A possible resolution for the observed clashes could be envisioned if the loop comprising CDR-H2, which contains many Gly residues, were to adopt a slightly different conformation. Alternately, if the rotamers for H-Arg-52, H-Arg-57, H-Tyr-59 and Aβ-Asp-7 were to be different, the modeled protein would be able to accommodate the conformation of the peptide illustrated and overcome the clashes. [file alzrt261-S3.pdf]
