# Supplementary material for: Crystal structure reveals conservation of amyloid-β conformation recognized by 3D6 following humanization to bapineuzumab
Source: Alzheimers Res Ther. 2014 Jun 2;6(3):31. doi: 10.1186/alzrt261 (PMC4095729; doi:10.1186/alzrt261)

Additional Figure 3. Interaction of framework residue with CDRs. VL residue V2 (thick purple stick in center) which was retained in bapineuzumab based on predicted interaction with residues in CDR-L1 and L3. L1 shown in dark blue and L3 in light blue. Hydrogen bonds are shown as dotted lines.

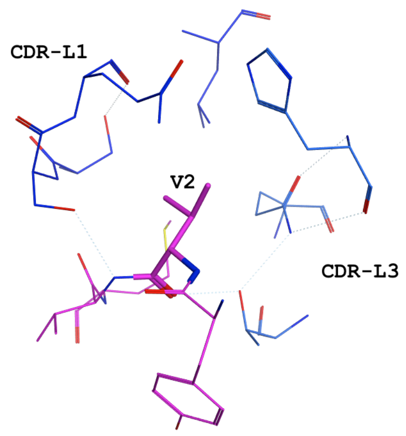

Supplement: Additional file 4: Figure S3 — A pdf file. Interaction of framework residue with CDRs. VL residue V2 (thick purple stick in center) which was retained in bapineuzumab based on predicted interaction with residues in CDR-L1 and L3. L1 shown in dark blue and L3 in light blue. Hydrogen bonds are shown as dotted lines. [file alzrt261-S4.pdf]
