# Supplementary material for: Crystal structure reveals conservation of amyloid-β conformation recognized by 3D6 following humanization to bapineuzumab
Source: Alzheimers Res Ther. 2014 Jun 2;6(3):31. doi: 10.1186/alzrt261 (PMC4095729; doi:10.1186/alzrt261)
Supplement: Additional file 5 — ‘Structural observations in 3D6 regarding mouse VL Fr residues retained during humanization, confirming predictions based on homology model.’ [file alzrt261-S5.docx]

Additional material:

*Structural observations in 3D6 regarding mouse VL Fr residues retained during humanization, confirming predictions based on homology model.*

As mentioned in main text, mouse VL Fr residues V2, S26 and Q27 were retained in bapineuzumab. Structural examination of 3D6 crystal reveals specifically Val2 makes contacts with Ser26 and Gln27 in L1, and His93 and Thr97 in L3, contributing to the conformation of these CDRs, most notably His93 being involved in binding to antigen (Table 3 & 4). This interaction is illustrated in supplementary figure 2. The other VL mouse Fr residues retained in bapineuzumab, Leu36 and Arg46 were predicted to be packing residues. The crystal structure of 3D6 Fab confirms inter-chain hydrophobic packing interactions <4Å involving Leu36 and VH Fr residues Trp103 and Leu45. Leu36 in VL is also observed to participate in intra-chain hydrophobic packing interactions with Trp89 in L3, which is a direct antigen-binding residue. VL residue-Arg46 is observed to participate in multiple inter-chain hydrogen bonds with hydroxyl residues from Ser99, Ser100a, Ser100b (main-chain), the latter two residues directly contacting antigen, and via a water molecule with Asp101 in CDR H3. Thus it maintains the conformation of this CDR as well as participating in inter-chain packing. It also forms an interaction via it e-amino group with Asp55 in CDR-L2, thus supporting the conformation of this CDR.

In similar fashion, three mouse VH framework residues were retained in humanized 3D6 VHv1 (Ala49, Val93, and Arg94) based on their contribution to preservation of CDR conformations in the homology model. These predictions were borne out in the crystal structure of 3D6. Specifically, Ala49 contacts Ser35 in H1, as well as Ser50, Tyr58, Tyr59 in H2, Ser50 and Tyr58 binding antigen. Likewise, V93 contacts Met34 in H1 and Tyr95 and Ser100b in H3, all directly contacting antigen, as well as Ser35 in H1, and Tyr102 in H3. Finally, Arg94 can be seen as a “ligand”, occupying a binding pocket comprised of side chains of CDR residues Tyr32 and M34 in H1, the latter binding antigen, and antigen binders Tyr95 and Ser100b in H3 as well as Asp96, Asp101 and Tyr102.
