# Supplementary material for: Crystal structure reveals conservation of amyloid-β conformation recognized by 3D6 following humanization to bapineuzumab
Source: Alzheimers Res Ther. 2014 Jun 2;6(3):31. doi: 10.1186/alzrt261 (PMC4095729; doi:10.1186/alzrt261)

Additional Figure 4. Surface plasmon resonance binding profiles of murine-3D6 IgG2b (red), chimeric-3D6 IgG1 (blue) and humanized-3D6 v2 (green) are shown on 5.0 RU of biotinylated-Aβ1-10 peptide immobilized on streptavidin coated chip.

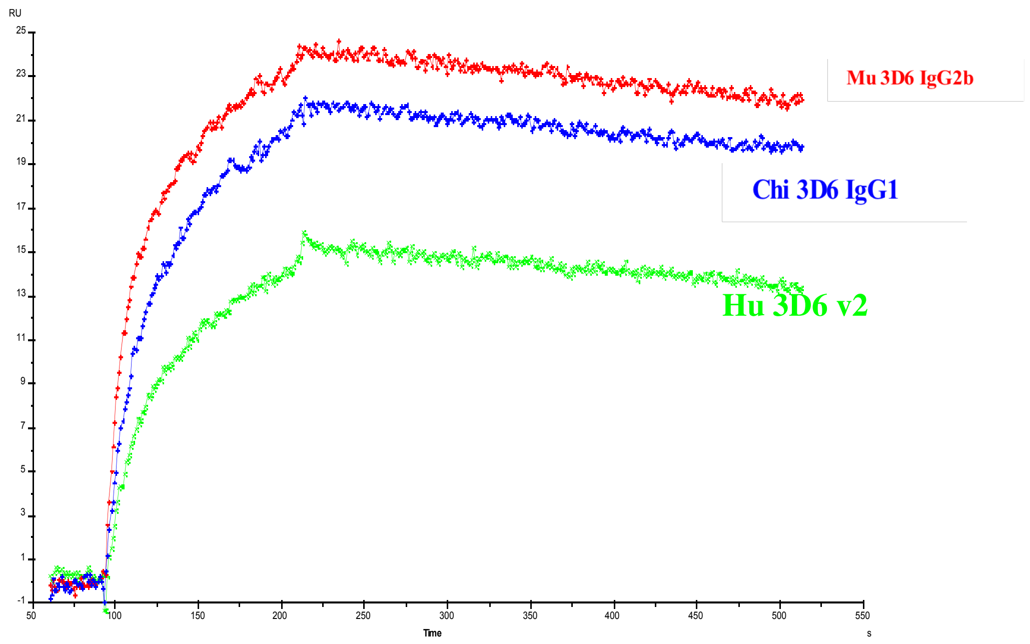

Supplement: Additional file 6: Figure S4 — A pdf file. Surface plasmon resonance binding profiles of murine-3D6 IgG2b (red), chimeric-3D6 IgG1 (blue) and humanized-3D6 v2 (green) are shown on 5.0 RU of biotinylated-Aβ1-10 peptide immobilized on streptavidin coated chip. [file alzrt261-S6.pdf]
